# Detailed Speciation of Semi-Volatile and Intermediate-Volatility Organic Compounds (S/IVOCs) in Marine Fuel Oils Using GC × GC-MS

**DOI:** 10.3390/ijerph20032508

**Published:** 2023-01-31

**Authors:** Rongzhi Tang, Kai Song, Yuanzheng Gong, Dezun Sheng, Yuan Zhang, Ang Li, Shuyuan Yan, Shichao Yan, Jingshun Zhang, Yu Tan, Song Guo

**Affiliations:** 1 School of Energy and Environment, City University of Hong Kong, Kowloon 999077, Hong Kong, China; 2 Shenzhen Research Institute, City University of Hong Kong, Shenzhen 518057, China; 3 School of Environment and Materials Engineering, Yantai University, Yantai 264003, China; 4 State Key Joint Laboratory of Environmental Simulation and Pollution Control, International Joint Laboratory for Regional Pollution Control, Ministry of Education (IJRC), College of Environmental Sciences and Engineering, Beijing 100871, China; 5 China Automotive Technology and Research Center (CATARC), Beijing 100176, China; 6 Department of Investigation Shanghai Police College, Shanghai 200137, China; 7 School of Chemical Engineering and Technology, Sun Yat-sen University, Zhuhai 519082, China

**Keywords:** marine fuel oil, semi-volatile organic compounds, intermediate-volatility organic compounds, comprehensive two-dimensional gas chromatography (GC × GC)

## Abstract

Ship emissions contribute substantial air pollutants when at berth. However, the complexity and diversity of the marine fuels utilized hinder our understanding and mapping of the characteristics of ship emissions. Herein, we applied GC × GC-MS to analyze the components of marine fuel oils. Owing to the high separation capacity of GC × GC-MS, 11 classes of organic compounds, including *b*-alkanes, alkenes, and cyclo-alkanes, which can hardly be resolved by traditional one-dimensional GC-MS, were detected. Significant differences are observed between light (-10# and 0#) and heavy (120# and 180#) fuels. Notably, -10# and 0# diesel fuels are more abundant in *b*-alkanes (44~49%), while in 120# and 180#, heavy fuels *b*-alkanes only account for 8%. Significant enhancement of naphthalene proportions is observed in heavy fuels (20%) compared to diesel fuels (2~3%). Hopanes are detected in all marine fuels and are especially abundant in heavy marine fuels. The volatility bins, one-dimensional volatility-based set (VBS), and two-dimensional VBS (volatility-polarity distributions) of marine fuel oils are investigated. Although IVOCs still take dominance (62–66%), the proportion of SVOCs in heavy marine fuels is largely enhanced, accounting for ~30% compared to 6~12% in diesel fuels. Furthermore, the SVOC/IVOC ratio could be applied to distinguish light and heavy marine fuel oils. The SVOC/IVOC ratios for -10# diesel fuel, 0# diesel fuel, 120# heavy marine fuel, and 180# heavy marine fuel are 0.085 ± 0.046, 0.168 ± 0.159, 0.504, and 0.439 ± 0.021, respectively. Our work provides detailed information on marine fuel compositions and could be further implemented in estimating organic emissions and secondary organic aerosol (SOA) formation from marine fuel storage and evaporation processes.

## 1. Introduction

As a main component of PM, organic aerosol (OA) can both be directly emitted from the emission sources, namely primary organic aerosol (POA), and formed by multigenerational reactions of precursors, called secondary organic aerosol (SOA). While POA can be precisely measured and modeled, large discrepancies still exist between modeled and measured SOA. One of the key reasons is the missing measurements of semi-volatile and intermediate-volatility organic compounds (S/IVOCs). S/IVOCs represent a subset of organic compounds with effective saturation concentrations (C*) of 10^0^–10^2^ and 10^3^–10^6^ μg m^−3^, roughly equivalent to the volatility of C_23_-C_32_ and C_12_-C_22_ *n*-alkanes [1,2]. Laboratory studies have confirmed high SOA yields of typical S/IVOCs species, e.g., long-chain *n*- and *b*- and cycloalkanes [3,4,5], naphthalenes [6] and polycyclic aromatic hydrocarbons (PAHs) [7]. Source emission and field studies also indicate S/IVOCs are ubiquitous precursors for SOA formation and can contribute up to 90% of the modeled SOA [1,8,9,10,11,12,13,14,15,16]. Though great achievements have been made through the incorporation of S/IVOCs into SOA simulation models, gaps still exist between modeled and measured SOA [8].

Previously, S/IVOCs were analyzed using one-dimensional gas chromatography/mass spectrometry (GC/MS), with most of the S/IVOCs existing as co-eluting compounds, i.e., unresolved complex mixture (UCM) due to the exponential increase of the isomers as the retention time increases [17]. Speciated S/IVOCs, i.e., *n*-alkanes, some *b*-alkanes, and PAHs, only account for a small portion of the total S/IVOCs. Considering the large lump of the unspeciated S/IVOCs, Zhao et al. [1] proposed a semi-quantitative method to quantify the total S/IVOCs. The key idea is to divide unspeciated S/IVOCs into different bins according to their saturation vapor pressure (volatility) and apply the *n*-alkane response factor in the same bin to estimate their total mass. This *n*-alkane equivalent method has been widely used in the quantification of S/IVOCs mass in ambient measurements and source emission studies [9,11,14,18,19,20,21]. However, the lack of detailed speciation brought some uncertainties to the estimation results, which resulted in further uncertainties in the model simulation. More recently, comprehensive two-dimensional gas chromatography-mass spectrometry (GC × GC-MS) has been applied to identify and quantify more individual species and groups of compounds based on their similar chemical structures [22,23]. Liang et al. [22] utilized GC × GC-ToF-MS to study the aliphatic isomers of lubricants. Xu et al. [24,25] took full advantage of this technique to perform detailed S/IVOC analysis in ambient air. Alam et al. [23] used GC × GC-ToF-MS to map and quantify isomer sets of hydrocarbons in diesel fuel, lubricating oil, and diesel exhaust emissions and discovered that more than 75% of the mass can be reconstructed using this technique. They found that gas-phase organic emissions resembled diesel fuel, while the patterns of particulate matter were similar to lubricating oils. As a result, understanding the compositions of oils is crucial for implying the emission profiles of mobile sources.

With the rapid development of globalization and international trade, ship transportation has become an indispensable part of worldwide trade due to its low cost and high volume [26,27,28]. Ship emission from inland rivers, seaports, and straits is becoming an increasingly important anthropogenic source for coastal and riverine cities [29,30]. Atmospheric ship emissions, including SO_2_, NO_x_ (NO + NO_2_), hydrocarbons, and particulate matter (PM), can greatly influence human health, as well as the regional and global atmosphere [31]. Field measurements and model studies indicate that ship emissions have substantial contributions to air quality in coastal areas [32,33,34]. For example, Liu et al. [35] discovered that ship emissions could contribute to 20–30% of the total PM_2.5_ within tens of kilometers of coastal and riverside Shanghai during ship-plume-influenced periods. Coincidentally, a study around the port of Gothenburg reported that local and regional shipping can contribute substantially to areas around city ports, where local and regional shipping contributed to 14% and 26% for NO_2_, 2.2%, and 10.3% for PM_2.5_, respectively [36]. More recently, researchers started to study the S/IVOCs emissions from ships. Huang et al. [14] determined fuel-based emission factors (EFs) of a large cargo vessel and found high IVOCs EFs (1003 ± 581 mg kg fuel^−1^), which is a magnitude higher than that of the gasoline vehicles [9]. The authors also highlight the importance of fuel type in determining ship IVOC emissions, volatility distribution, and SOA production. Lou et al. [20] investigated IVOCs from a ship’s main engine burning heavy fuel oil and found IVOCs EFs of 20.2–201 mg kg fuel^−1^. Su et al. [19] considered that ship auxiliary engine burning waste cooking oil could reduce 50% of IVOC emissions compared to that of marine gas oil. They also find that the compositions and volatility distributions of exhaust IVOCs varied from that of unburned fuel. Lu et al. [12] emphasized that the fuel type was the predominant factor that influenced the volatility distribution of sources. As numerous diesel-powered ongoing and offshore ships vary in fuel types and other oils, considering the complexity and diversity of the marine oils used, it is an urgent need to map and quantify the chemical compositions of the oils used in marine ships.

Due to the rapid increase in ship emissions, the International Maritime Organization (IMO), a specialized agency of the United Nations, has formulated regulations to regulate pollution from ships. The new IMO 2020, which came into force on 1 January 2020 limits the sulfur in the fuel oil used on board ships operating outside designated emission control areas to 0.5% m/m, which is a significant reduction from the previous limit of 3.5% [37]. Though great achievements have been made in the analysis of marine fuel oils in the past years, no relevant speciation and composition information can be found for marine fuel oils after 2020. Despite being limited by the analytical techniques, previous studies did not provide the volatility and polarity distribution of marine fuel oils, which may limit our understanding of marine fuel oils and thus their environmental impact.

In the present work, a comprehensive two-dimensional gas chromatography-quadrupole mass spectrometry (GC × GC-qMS) was applied to quantify and characterize eight marine fuel oils, which were collected after the implementation of IMO 2020. Their chemical compositions, volatility, and polarity distributions were provided.

## 2. Material and Methods

### 2.1. Tested Marine Fuel Oils

Eight kinds of marine fuel oils were collected before combustion, i.e., they are fresh and not used. Fuels include 0#, -10#, 120#, and 180# which are mainly based on their physicochemical properties, e.g., density and viscosity. Notably, 0# and -10# are mainly used for high-speed diesel engines, while 120# and 180# are used for medium- and low-speed marine ship engines. Note that the numbers 0#, -10#, 120#, and 180# are the common names in China, which correspond to DMX, DMA, DMX, RMD 15, and RME 25 by international criteria, respectively. To simplify the comparison, we will use common names afterward.

Detailed information on the collected marine oils is shown in Table 1.

### 2.2. Analytical Instrument and Data Analysis

The fuel samples were analyzed via a GC × GC-qMS system (GC-MS-TQ8050, Shimadzu, Japan). Notably, 1 uL of the diluted fuel sample (10 μL oil in 1 mL *n*-hexane) was directly injected into the inlet and evaporated at 280 °C. The evaporated organics were split into the column at a ratio of 15:1 and a flow rate of 1.0 mL/min (He). The column temperature was held at 50 °C for 5 min and ramped to 250 °C at a rate of 5 °C/min followed by an isothermal hold for 5 min, after which the temperature increased to 280 °C at 10 °C/min and held for 20 min. The first and second capillary columns used in the GC × GC system were SH-Rtx-5 ms (30 m × 0.25 mm × 0.25 μm) and SGE BPX-50 (2.5 m × 0.1 mm × 0.1 μm). A dual-stage modulator (ZOEX, Houston, TX, USA) was used to transfer the analytes from the first dimension to the second dimension. The modulation time was 6 s with a thermal spray time of 350 ms. The separated analytes were then transferred into the MS detector through an interface at 280 °C. The ion source temperature was set at 200 °C with an electron impact ionization energy of 70 eV. The MS was operated in scanning mode from *m*/*z* 33 to 500 at a speed of 33 Hz. The attained MS spectra were then compared with the National Institute of Standard and Technology (NIST 17) library. GC Image v2.8r2 (Zoex Corporation, Houston, TX, USA) was applied to process the data.

**Figure 1:**
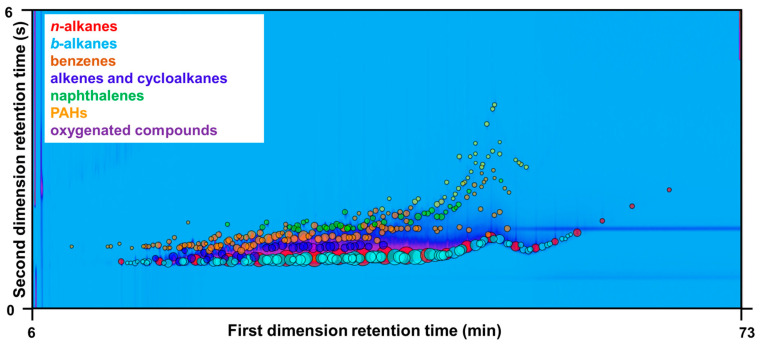
A typical chromatogram of 0# diesel fuel (No 1). The blob size is commensurate with its mass percentage. Column bleedings are excluded from the blobs.

Organics with similar structures are featured similar physicochemical properties, which can be utilized to separate different classes of organic compounds. Though the individual chemical compounds may vary, marine fuel oils share similar chemical groups. Therefore, a Computer Language for Identification of Chemicals (CLIC) qualifiers was set up and applied to match peaks and their fragmentation patterns. CLIC qualifiers are customer-built expressions that describe rules or constraints for selecting chemical peaks or data points based on multi-dimensional chromatographic properties and mass spectral features [38]. These customer-built CLIC qualifiers were then applied to distinguish specific classes of compounds with similar characteristics and incorporated with a blob-by-blob integration tool [39], as shown in Figure 1 as an example. Chromatograms of other different kinds of marine oils were shown in the supplement Figure S1. The CLIC expressions used in this study are summarized in Supplement Text S1.

The column combination in this work is non-polar SH-Rxi-1ms (1st) and mid-polar BPX50 (2nd). As a result, the first retention time is linked to volatility and the second retention time is related to polarity [40,41,42]. We slice the chromatograms into 2D bins accordingly. The bins in 1st retention times are called the volatility bins, with a decrease in volatility from B10 to B30 following the pipeline of previous studies [1,9,15]. The bins in 2nd retention times are cut by 0.5 s, with an increase of polarity from P1 to P12. The two-dimensional panel is constructed by the combination of 1D and 2D bins, i.e., the volatility and polarity properties of chemicals [39].

We apply pixel-based multiway principal component analysis (MPCA) to identify fingerprints of different marine fuel oils. MPCA has been demonstrated to be a useful tool to resolve compositional changes related to different sources [39,43]. Here, two-dimensional gas chromatograms were exported as net-work common data form (netCDF) files and loaded into RGC × GC toolbox [44]. The imported data were then pre-processed by performing smoothing and baseline correction, after which peak alignments were carried out following the two-dimensional correlation optimized warping (2DCOW) algorithm. The RGC × GC toolbox helps to unfold the chromatogram into a two-way matrix which can be further used for pixel feature extraction [39].

## 3. Results and Discussion

### 3.1. Chemical Compositions of the Organic Compounds in Different Marine Fuel Oils

**Figure 2:**
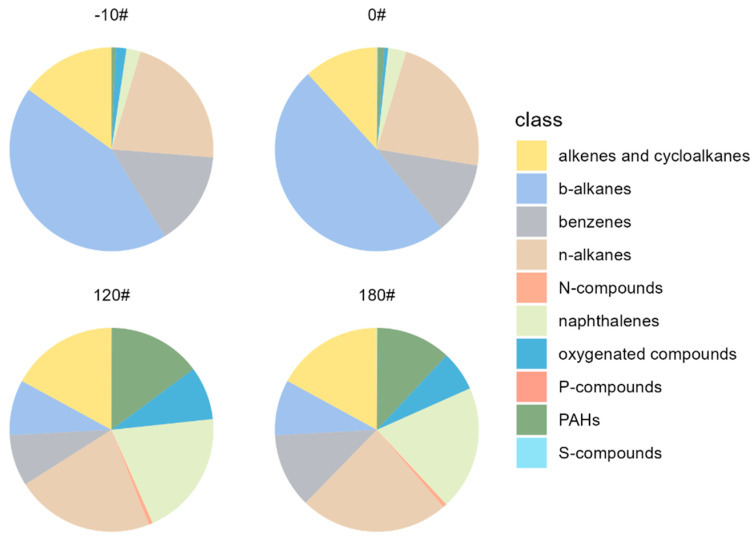
Chemical compositions of organic compounds in different types of fuel (-10#, 0#, 120#, and 180#).

The chemical compositions of the organic compounds grouped by fuel types are divided into different compositions, including alkenes and cycloalkanes, *b*-alkanes, benzenes, *n*-alkanes, nitrogen-containing compounds (N-compounds), naphthalenes, oxygenated compounds, phosphorus-containing compounds (P-compounds), PAHs, and sulfur-containing compounds (S-compounds). The mass spectrum of the different marine oils is characterized by sequences of C_n_H_2n+1_, C_n_H_2n_, C_n_H_2n-1,_ and C_n_H_2n-3_. Mass fragments of *m*/*z* 57 (C_4_H_9_^+^), *m*/*z* 69 (C_5_H_9_^+^), and *m*/*z* 82 and 83 (C_6_H_10_^+^ and C_6_H_11_^+^) are predominant in the whole spectrum, indicating the high abundance of straight, branched or cycloalkanes. On average, aliphatic compounds, i.e., alkenes and cycloalkanes, *b*-alkanes, and *n*-alkanes, account for 70.1% of marine fuel oils. Benzenes and naphthalenes account for 12.1% and 9.1%, while other PAHs contribute 5.4%. However, regarding different types of fuels, marine oils diverge from each other significantly (Figure 2). A significant difference is observed between light (-10# and 0#) and heavy (120# and 180#) fuels. Notably, -10# and 0# diesel fuels are more abundant in *b*-alkanes (44~49%), while in 120# and 180#, heavy fuels *b*-alkanes only account for 8%. Significant enhancement of naphthalene proportions is observed in heavy fuels (20%) compared to diesel fuels (2~3%). Naphthalenes are mainly observed in heavy fuel oils, indicating that when evaluating storage and evaporative loss of marine oils, naphthalenes could be utilized to mark the heavy marine fuel oil emissions.

Furthermore, pixel-based MPCA is utilized to characterize similarities and differences (fingerprints) between different marine fuel oils, as shown in Figure S2. Just like the molecular compositions mentioned above, the differences between fuel compositions could be largely explained by *b*-alkanes and IVOC *n*-alkanes (mainly C_12_–C_22_). Similarities of chromatograms from the pixel-based level could be explained by SVOC *n*-alkanes (mainly C_23_–C_31_), naphthalenes and other PAHs, and hopanes (such as 28-Nor-17alpha(H)-hopane). In atmospheric implication of organic emissions from marine fuel evaporation, IVOC alkanes (especially *b*-alkanes) are of special concern in distinguishing light and heavy fuel emissions.

### 3.2. Volatility and Polarity Distribution of Marine Fuel Oil

**Figure 3:**
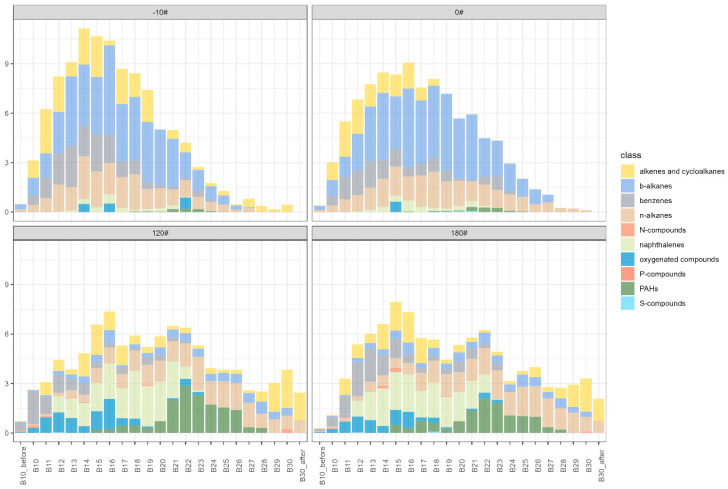
Average volatility distribution of different types of marine fuel oils (-10#, 0#, 120#, and 180#).

Volatility distribution is considered one of the important factors that influence SOA formation. However, the volatility distributions of the total organic compounds for different kinds of marine oils are poorly understood. Figure 3 shows the average volatility distribution of the characterized organic compounds from different types of marine fuel oils. A transparent unimodal distribution can be found in the diesel fuels (-10# and 0#), with peak concentrations occurring at B14–B16 (IVOC range). The majority of the species lie in the IVOCs range, accounting for more than 78% of the total organic mass in -10# and 0# diesel fuels (Figure S3). Even though diesel fuel compositions are indeed similar in volatility distributions, slight differences could also be found. The variation of B10–B20 (VOC-IVOC range) species in -10# diesel is larger than in 0# diesel, causing a sharper peak, mainly derived from *b*-alkane contributions. Heavy marine fuel oils display bimodal distributions, as shown in Figure 3. The volatility distribution of heavy fuels is much flatter than diesel fuels. Although IVOCs still take dominance (62–66%), the proportion of SVOCs in heavy marine fuels is largely enhanced, accounting for ~30% compared to 6~12% in diesel fuels. The molecular compositions of heavy marine fuels are also different from diesel fuels in each volatility bin. However, the constituents of 120# and 180# fuels are rather similar. Naphthalenes (B12–B22, IVOC range) and other PAHs (B20–B26, IVOC-SVOC range) are extensively detected in heavy fuels, while the contents of *b*-alkanes in light fuel are much higher than heavy fuels across the IVOC-SVOC range. Cycloalkanes (especially hopanes) in heavy fuels are also more extensively detected than light diesel fuels in B28–B31 (SVOC range).

**Figure 4:**
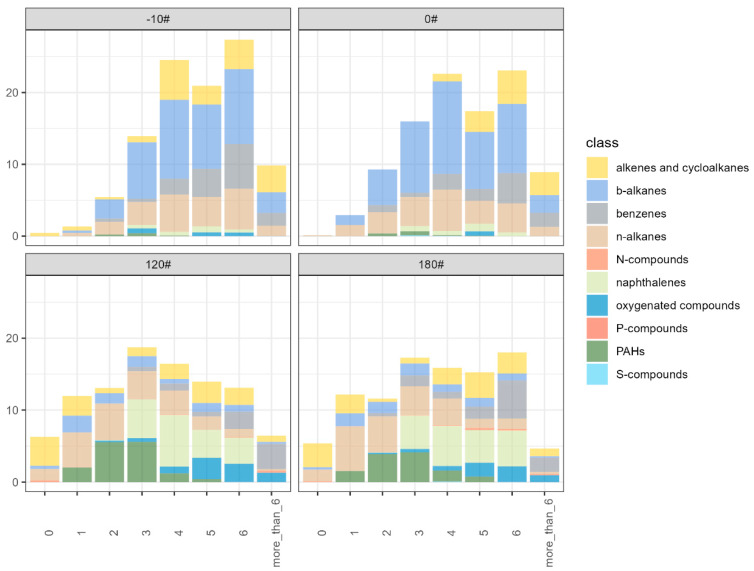
Average volatility distribution of organic emissions for -10#, 0#, 120#, and 180# marine fuels based on the VBS model. The x-axis is the saturated vapor concentration (C*) in μg m^−3^.

Figure 4 shows the average volatility distribution of marine fuel oils based on the VBS model. VBS is a simplified model to illustrate the emission pattern of organic emissions which aggregates the organic emission factors in each VBS volatility bin. VBS could be further implemented in SOA estimation with a comparison of gasoline and diesel emissions. Additionally, the volatility bins in Figure 3 are rather complicated in the model simulation. The volatility peaks of diesel fuels (0# and -10#) occur at C* (saturated vapor concentration) =10^4^ and 10^6^ μg m^−3^, with large contributions from *b*-alkanes and *n*-alkanes. The VBS volatility distribution of heavy fuels is much flatter than diesel fuels, with ~10% in each 10^1^~10^6^ μg m^−3^ VBS bin in the IVOC-SVOC range. As mentioned above, hopanes in 10^0^ (1) μg m^−3^ VBS bins in heavy fuels are much more abundant than diesel fuels, although hopanes could also be detected in 0# and -10# diesel fuels.

**Figure 5:**
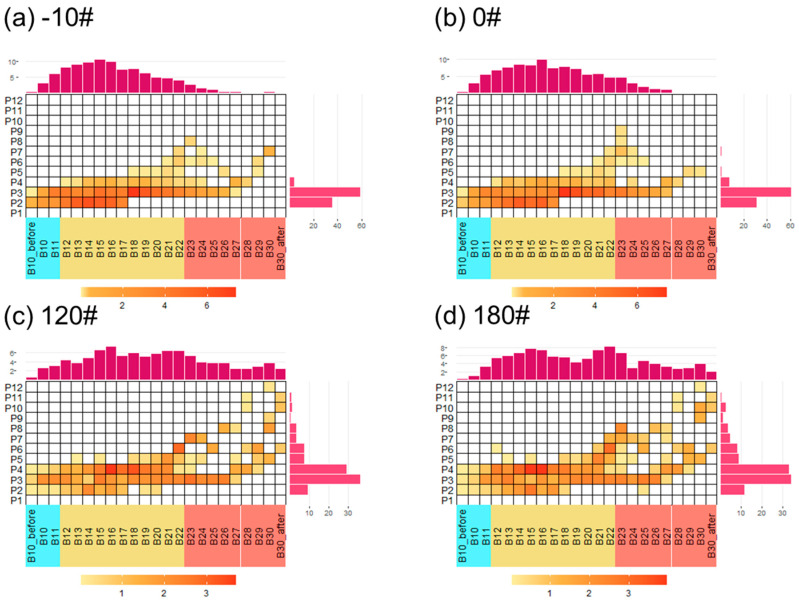
Average volatility-polarity distribution of (**a**) -10#, (**b**) 0#, (**c**) 120#, and (**d**) 180# marine fuel oils. Blue, orange, and red color blocks represent VOCs, IVOCs, and SVOCs with decreasing volatility. Polarity distribution was shown on the y-axis, with increasing polarity from P1–P12. Note that the values shown here all represent mass percentages.

We further apply a novel two-dimensional panel [45] that incorporates the volatilities and polarities to investigate the physicochemical properties of the fuels, as shown in Figure 5. The VBS model simplified the SOA simulation by aggregating organics in the same volatility bin, causing uncertainties due to the SOA yields of organics in the same volatility bin are sometimes much different (such as PAHs and alkanes). Volatility-polarity distributions could be further implemented in the two-dimensional VBS model for a better simulation of SOA. By applying different SOA yields for organics in different volatility-polarity bins, the SOA simulation could be simplified with a better estimation compared to the one-dimensional VBS model. As shown in Figure 4 and Figure 5, the volatility-polarity distributions and the one-dimensional volatility distribution of -10# and 0# diesel fuels are quite similar, with slight differences in chemical compositions. This indicates that when evaluating organics from marine diesel fuel emissions (such as fuel evaporation), the influencing factor of diesel fuel type may have less impact on the VBS SOA simulation. The variance of heavy marine fuels is larger than diesel fuels, either in volatility or polarity distribution. For instance, 180# fuel contains more polar compounds than 120# fuel (Figure 5). However, similarities in heavy fuel compositions are more predominant than differences, illustrating that simplification (VBS or 2D VBS) could also be applied in evaluating heavy fuel emissions regardless of heavy fuel type.

### 3.3. Metrics to Identify the Pollution Sources

As limited studies have been performed on detailed speciation of S/IVOCs, it is still an open question for researchers to distinguish different sources of S/IVOCs. Lu et al. [12] highlight the importance of attributing different emission exhausts to different source profiles to improve the simulation of SOA formation potential. Here, we use the SVOC-to-IVOC ratio as a metric to identify similarities and differences between various sources. In this work, the SVOC/IVOC ratios for -10# diesel fuel, 0# diesel fuel, 120# heavy marine fuel, and 180# heavy marine fuel are 0.085 ± 0.046, 0.168 ± 0.159, 0.504, and 0.439 ± 0.021, respectively. Notably, 0# diesel fuel contains more IVOC content compared to -10# diesel, due to a larger proportion of *b*-alkanes in B23–B26 (SVOCs). The SVOC/IVOC ratios for diesel fuels are significantly lower than for heavy marine fuels. Our study adds more information to marine oil fuel SVOC/IVOC ratios, which are lacking in previous work. We also provide an identification approach for the apportionment of marine fuel-related sources (such as marine fuel storage and evaporative emissions). The SVOC/IVOC ratios in this work are comparable with previous work. Previous studies have found SVOC/IVOC of 0.03–1.96, with an average value of 0.33 for US gasoline vehicles manufactured in different model years [15]. Except for one after-treatment-equipped diesel vehicle, the SVOC/IVOC ratios lay in a relatively narrow range, from 0.03 to 0.49 [16], which is similar to the ratios for marine fuels in this work. Huang et al. [14] found that the mean SVOC/IVOC ratios for a tested large cargo ship fueled with low-sulfur fuel (LSF) and high-sulfur fuel (HSF) were 0.23 and 0.96. Alam et al. [23] discovered that the SVOC/IVOC ratio for diesel exhaust particles is 1.42. Further work should be taken into account for the investigation of other types of marine fuels, as we only analyzed four marine fuels. We also recommend a comparison between GC × GC-MS and other instruments for a better understanding of marine fuel compositions.

## 4. Conclusions

Eight marine fuel oils were analyzed using GC × GC-MS. Notably, 11 classes of organics were identified owing to the high separation capacity of GC × GC-MS. On average, aliphatic compounds, i.e., alkenes and cycloalkanes, *b*-alkanes, and *n*-alkanes, account for 70.1% of marine fuel oils. Light diesel fuel (-10# and 0#) contained much more *b*-alkanes while heavy marine fuels (120# and 180#) are more abundant in naphthalenes and other PAHs. Hopanes are detected in all marine fuels and are especially abundant in heavy marine fuels. We also examine the volatility distribution of marine fuel organics in volatility bins, one-dimensional VBS, and two-dimensional VBS (volatility-polarity distributions). Diesel fuels (-10# and 0#) are predominant in the IVOC range (>78%), while SVOCs are enhanced in heavy fuels (~30%) compared to light fuels (~10%). Furthermore, the SVOC/IVOC ratio could be applied to tell between light and heavy marine fuel oils. The SVOC/IVOC ratios for -10# diesel fuel, 0# diesel fuel, 120# heavy marine fuel, and 180# heavy marine fuel are 0.085 ± 0.046, 0.168 ± 0.159, 0.504, and 0.439 ± 0.021, respectively.

## Figures and Tables

**Table 1:** Detailed information on the collected marine fuel oils. Note that all the fuels were used for the main engine.

No	Fuel Number (Chinese Common Name)	International Criteria	Density kg/m^3^	Kinematic Viscosity mm^2^/s	Sulfur Content (%)
1	0#	DMA	844	5.5	0.0008
2	-10#	DMX	830	6.2	0.005
3	-10#	DMX	824	5.8	0.008
4	120#	RMD	985	103.5	0.3
5	180#	RME	991	152.4	0.4
6	180#	RME	992	155.2	0.4
7	0#	DMA	842	3.9	0.0004
8	0#	DMA	839	4.6	0.0007

## Data Availability

The datasets used or analysed during the current study are available from the corresponding author (rongtang@cityu.edu.hk) on reasonable request.

## Supplementary Materials

The following supporting information can be downloaded at: https://www.mdpi.com/article/10.3390/ijerph20032508/s1, Text S1: Computer Language for Identifications of Chemicals (CLIC qualifiers); Figure S1: Chromatograms of the samples collected in this study, except for Sample No1, which has been shown in Figure 1. The blob size is proportional to its mass percentage. Column bleedings have been excluded from the blobs; Figure S2: (a) MPCA positive loading of the tested marine fuel oils, indicating the key species responsible for the similarities of the fuel samples; (b) MPCA negative loading of the tested marine fuel oils, indicating the key species responsible for the differences of the fuel samples; Figure S3: Resolved chemical species in different fuels based on volatilities.
