# Supplementary material for: Detailed Speciation of Semi-Volatile and Intermediate-Volatility Organic Compounds (S/IVOCs) in Marine Fuel Oils Using GC × GC-MS
Source: Int J Environ Res Public Health. 2023 Jan 31;20(3):2508. doi: 10.3390/ijerph20032508 (PMC9916049; doi:10.3390/ijerph20032508)
Supplement: Supplementary file 1 [file ijerph-20-02508-s001.zip › ijerph-2070057-SI.pdf]

Supporting Information:

Detailed Speciation of Semi-Volatile and Intermediate-Volatility Organic Compounds (S/IVOCs) in Marine Fuel Oils Using GC × GC-MS

Rongzhi Tang [1,2,3,*,†], Kai Song [4,†], Yuanzheng Gong [4,*], Dezun Sheng [3], Yuan Zhang [4], Ang Li [5], Shuyuan Yan [5], Shichao Yan [5], Jingshun Zhang [6], Yu Tan [7] and Song Guo [4]

1 School of Energy and Environment, City University of Hong Kong, Kowloon 999077, Hong Kong, China
2 Shenzhen Research Institute, City University of Hong Kong, Shenzhen 518057, China
3 School of Environment and Materials Engineering, Yantai University, Yantai 264003, China
4 State Key Joint Laboratory of Environmental Simulation and Pollution Control, International Joint Laboratory for Regional Pollution Control, Ministry of Education (IJRC), College of Environmental Sciences and Engineering, Beijing 100871, China
5 China Automotive Technology and Research Center (CATARC), Beijing 100176, China
6 Department of Investigation Shanghai Police College, Shanghai 200137, China
7 School of Chemical Engineering and Technology, Sun Yat-sen University, Zhuhai 519082, China
* Correspondence: rongtang@cityu.edu.hk (R.T.); xtgongyzh@126.com (Y.G.)
† These authors contributed equally to this work.

Table of Contents

Text S1. Computer Language for Identifications of Chemicals (CLIC qualifiers).

Figure S1. Chromatograms of the samples collected in this study, except for Sample No1, which has been shown in Figure 1. The blob size is proportional to its mass percentage. Column bleedings have been excluded from the blobs.

Figure S2. (a) MPCA positive loading of the tested marine fuel oils, indicating the key species responsible for the similarities of the fuel samples; (b) MPCA negative loading of the tested marine fuel oils, indicating the key species responsible for the differences of the fuel samples.

Figure S3. Resolved chemical species in different fuels based on volatilities.

Text S1. Computer Language for Identifications of Chemicals (CLIC qualifiers)

C3-Alkyl Benzenes

-((ordinal(91)-1)&(ordinal(120)<-3)&(retention(1)<30))((ordinal(105)-1)&(ordinal(120)<-3)&(r ctention(1)<30));

C4-Alkyl

Benzenes-((ordinal(91)-1)&(ondinal(134)<-3)(ordinal(105)-1)&(ordinal(134)<-3)&(retentionl

1)<80)((ordinal(119)-1)&(ordinal(134)<-3));

C5-Alkyl

Benzenes-((ordinal(92)-1)&(ordinal(148)<-3)X(ordinal(105)-1)&(ordinal(148)<-4)((ordinal(11 9)-

1)&(ordinal(148)<-4)((ordinal(133)-1)&(ordinal(148)<-3));

C6-Alkyl

Benzenes(fordinal(92)-1)&(ordinal(162)<-4)(ordinal(105)-1)&(ordinal(162)<-5)(ordinal(11 9)-1)&(ordinal(162)<-

5)((ordinal(133)-1)&(ordinal(162)<-5)((ondinal(147)<-2)&(ordinal(162)<-5)&(retention(2)<6)(ordinal(106)-

1)&c(ordinal(162)<-4))X(ordinal(91)-1)&(ordinal(162)<-4)

C7-Alkyl

Benzenes-((ordinal(105)-1)&(ondinal(176)<-4)((ordinal(133)-1)&(ordinal(176)<-4)((ordinal(1 61)-

1)&(ordinal(176)<-4)&(Retention(2)<5.5))(ordinal(91)-1)&(ordinal(176)<-3))((ordinal(147)-1)&(ordinal(119)<-

4)&(ordinal(176)<-4))(ordinal(106)-1)&(ordinal(176)<-4)X(ordinal(119) -1)&(ordinal(176)<-5));

Cl-Alkyl NAP-(ordinal(141)<-3)&(ordinal(115)<-3)&(ordinal(142)<-3); C2-Alkyl

NAP-((ordinal(141)-1)&(ondinal(156)-2)&(ordinal(115)-3)&(relative(141)>90)&(relativc(156)>5

0))((ordinal(156)-1)&(ordinal(141)-2)&(ordinal(155)-3)&(relative(156)-90)&(relative(141)-50)) C3-Alkyl NAP

-((ordinal(155)-1)&(ordinal(170)-2)&(Relativo(155)-80)&(Relativc(170)>25)((ordinal(170)-1) &(ondinal(155)-

2)&(Relative(170)-S0)&(Relative(155)p-60))(ordinal(141)-1)&(ordinal(170)-2) &(ordinal(115)-

3)&(Relative(141)>80)&(Relative(170)>25)&(Relative(115)>15))

C4-Alkyl

NAP-((ordinal(155)-1)&(ordinal(184)-2)&c(Relative(155)>80)&(Relative(184)>15)(ordinal(141)-

1)&(ordinal(184)-2)&(Relative(141)>80)&:(Relative(184)>15)((ondinal(169)-1)&(ordinal(184) -

2)&(Relative(169)-80)&(Relativc(184)30)(ordinal(184)-1)&(ordinal(169)-

2)&(Relative(184) >80)&(Relative(169)>50));

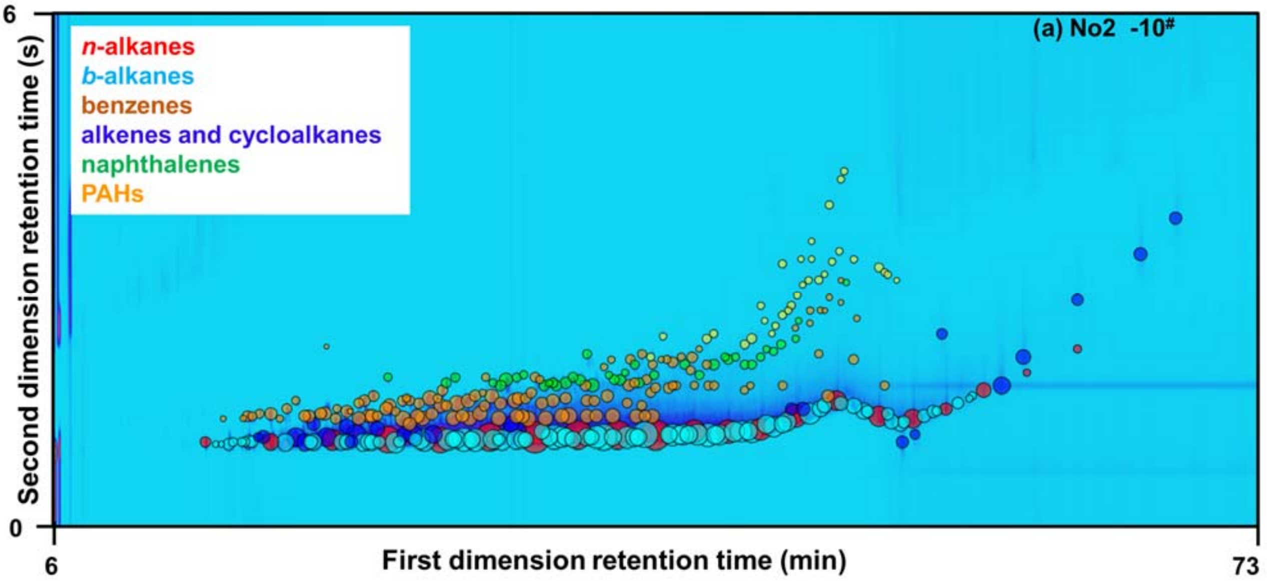

(a) No2 -10#
n-alkanes
b-alkanes
benzenes
alkenes and cycloalkanes
naphthalenes
PAHs
6
0
Second dimension retention time (s)
6
First dimension retention time (min)
73

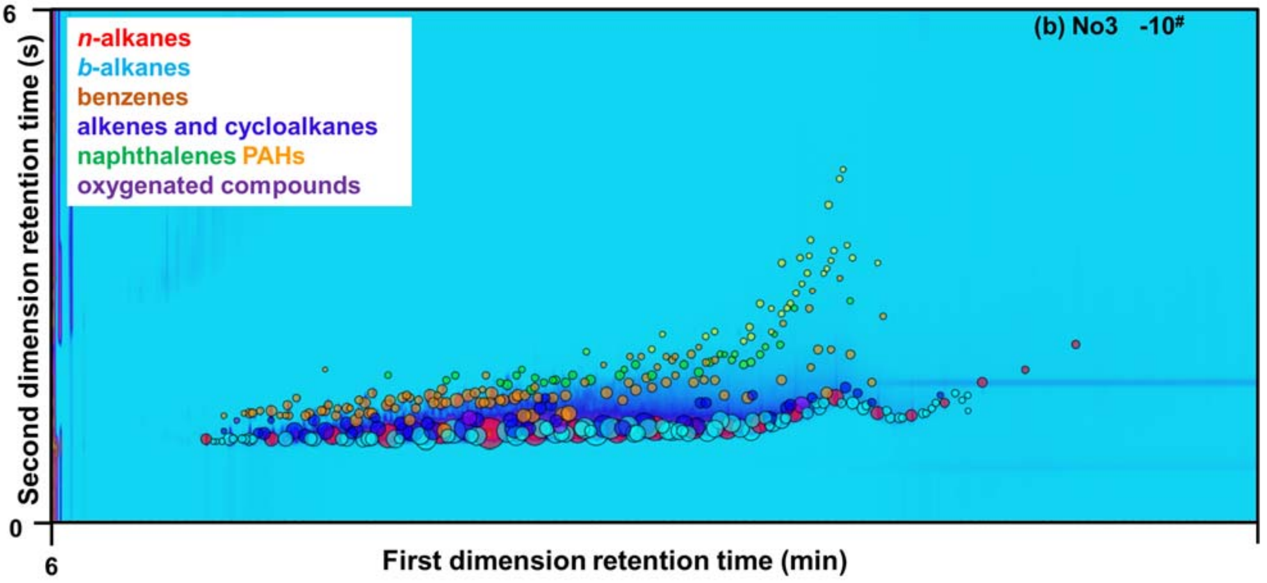

(b) No3 -10#
n-alkanes
b-alkanes
benzenes
alkenes and cycloalkanes
naphthalenes PAHs
oxygenated compounds
6
0
Second dimension retention time (s)
6
First dimension retention time (min)

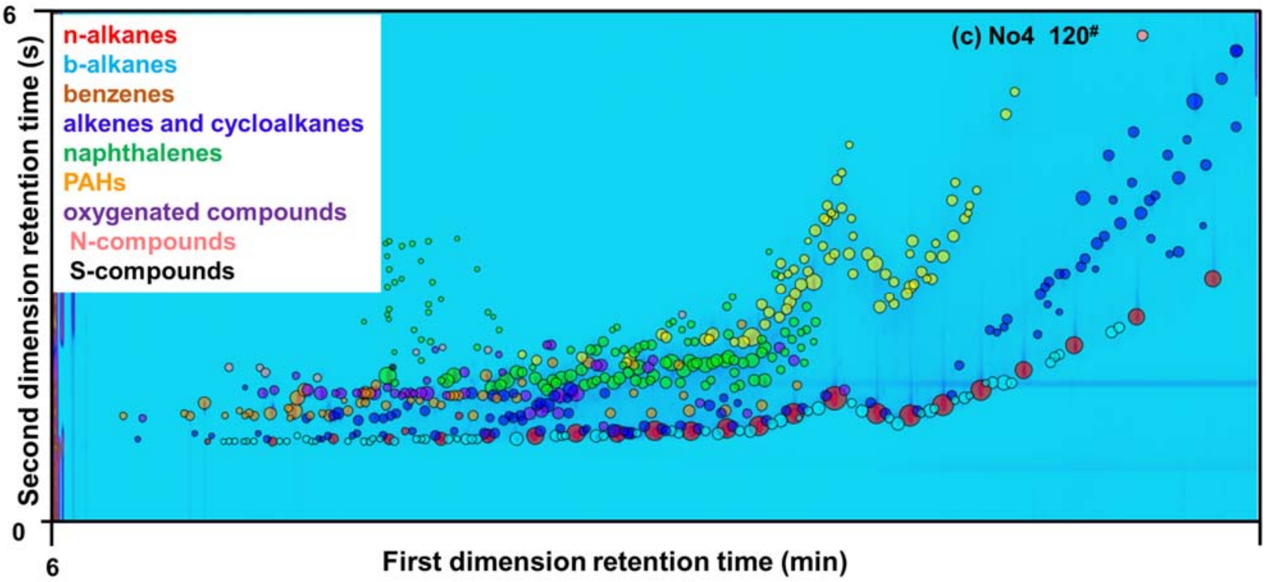
n-alkanes
b-alkanes
benzenes
alkenes and cycloalkanes
naphthalenes
PAHs
oxygenated compounds
N-compounds
S-compounds
(c) No4 120#
Second dimension retention time (s)
First dimension retention time (min)
6
0
6

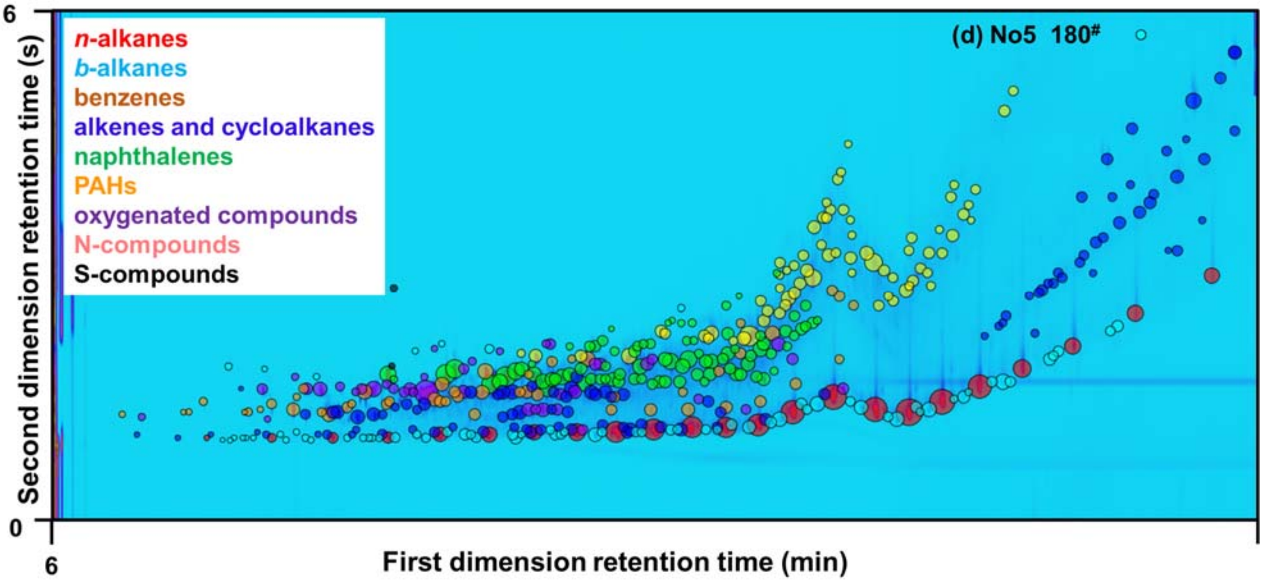
n-alkanes
b-alkanes
benzenes
alkenes and cycloalkanes
naphthalenes
PAHs
oxygenated compounds
N-compounds
S-compounds
(d) No5 180#
Second dimension retention time (s)
First dimension retention time (min)
6
0
6

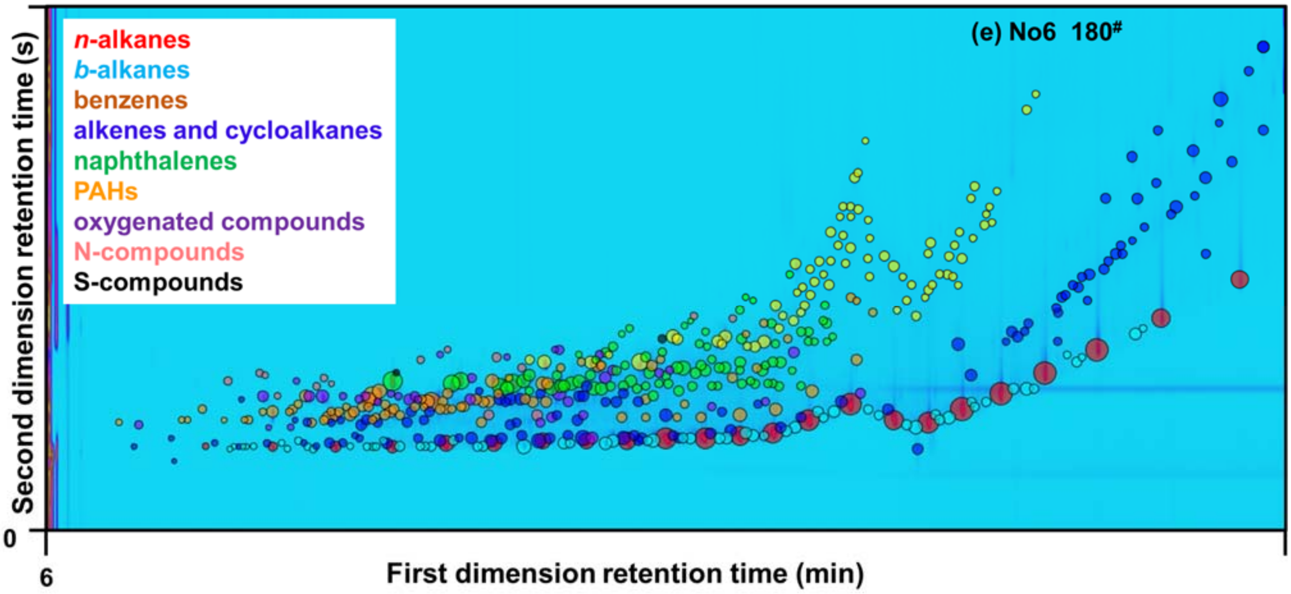

n-alkanes
b-alkanes
benzenes
alkenes and cycloalkanes
naphthalenes
PAHs
oxygenated compounds
N-compounds
S-compounds
(e) No6 180#
Second dimension retention time (s)
0
6
First dimension retention time (min)

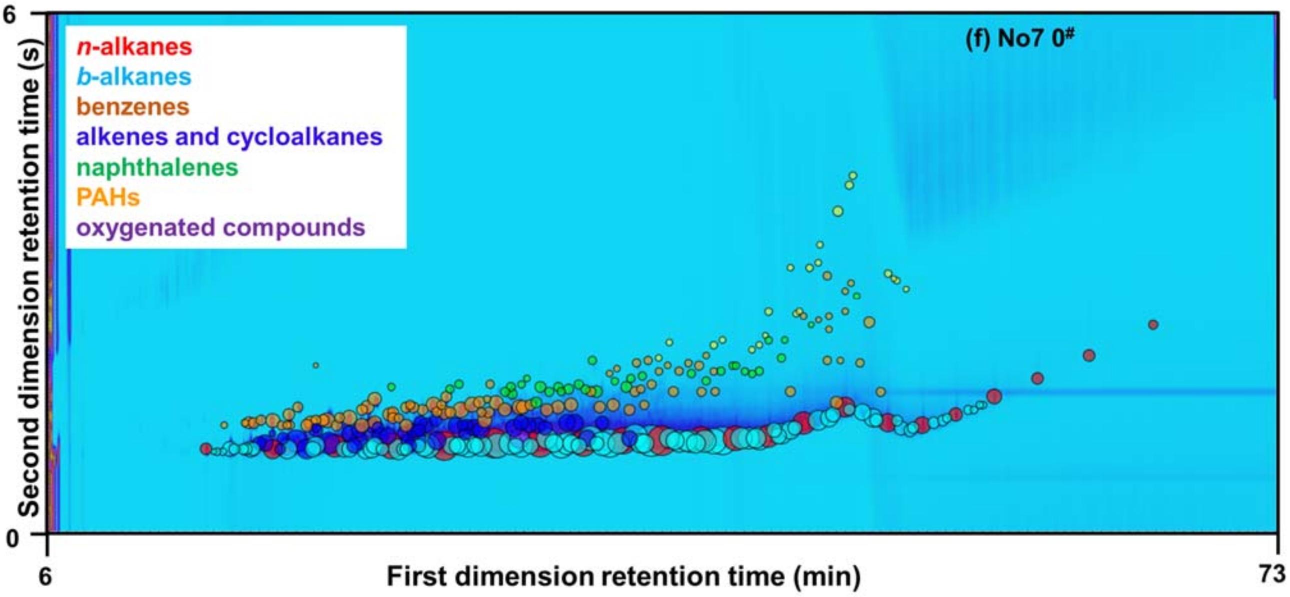

n-alkanes
b-alkanes
benzenes
alkenes and cycloalkanes
naphthalenes
PAHs
oxygenated compounds
(f) No7 0#
Second dimension retention time (s)
6
0
6
First dimension retention time (min)
73

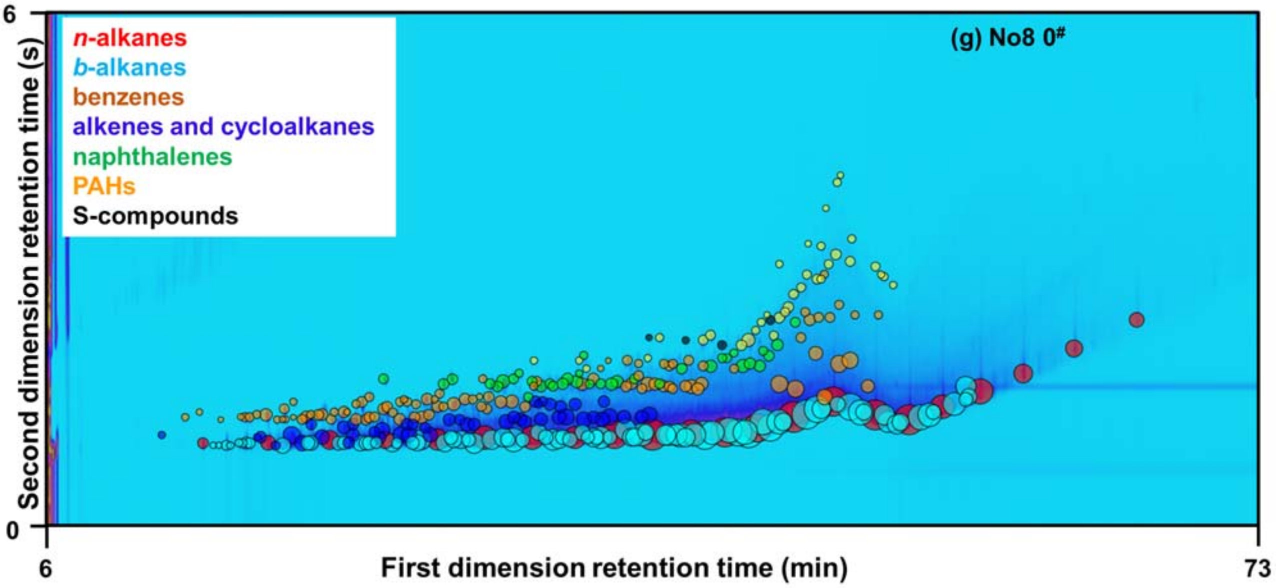

Figure S1. Chromatograms of the samples collected in this study, except for Sample No1, which has been shown in Figure 1. The blob size is proportional to its mass percentage. Column bleedings have been excluded from the blobs.

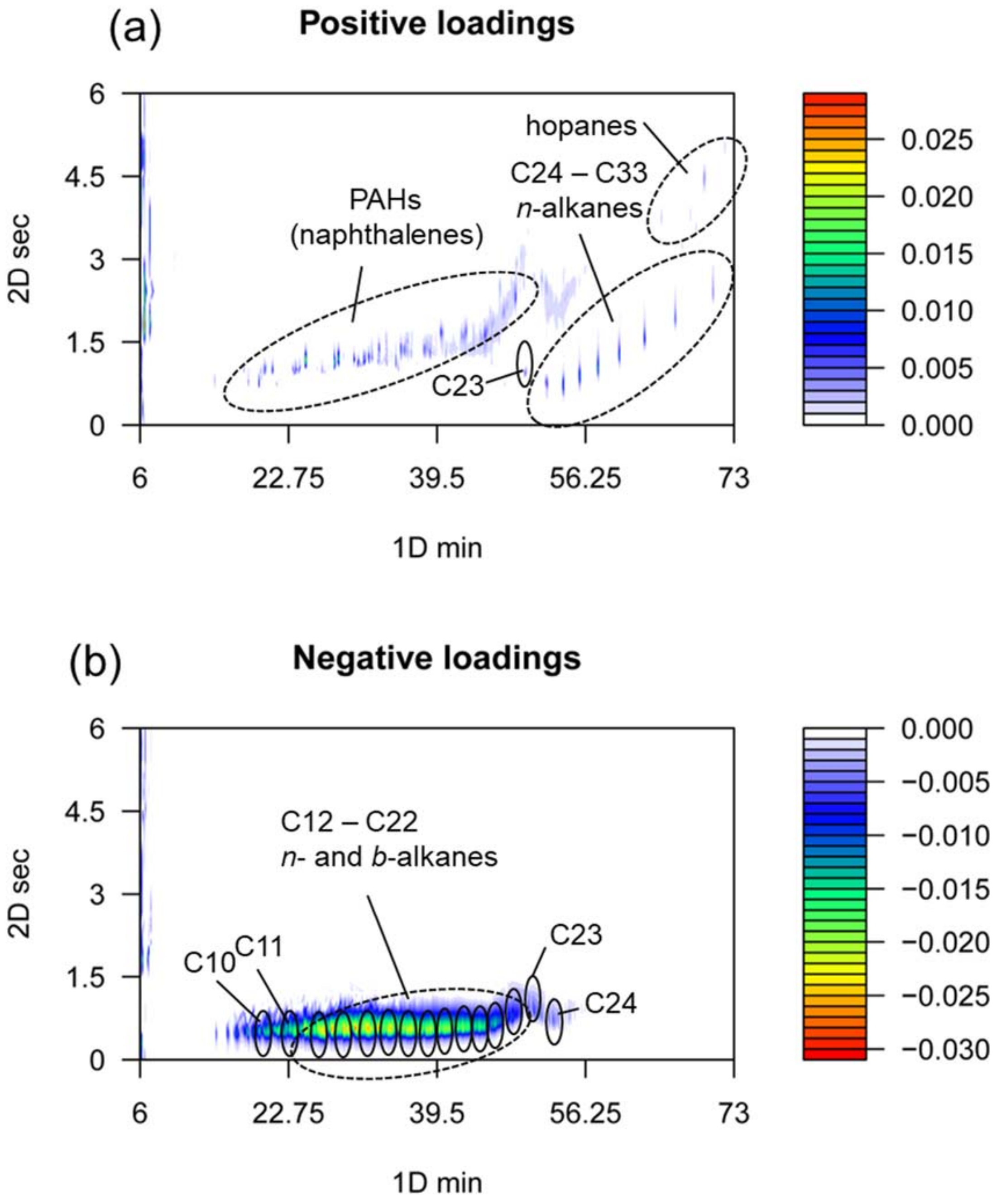

Figure S2. (a) MPCA positive loading of the tested marine fuel oils, indicating the key species responsible for the similarities of the fuel samples; (b) MPCA negative loading of the tested marine fuel oils, indicating the key species responsible for the differences of the fuel samples.

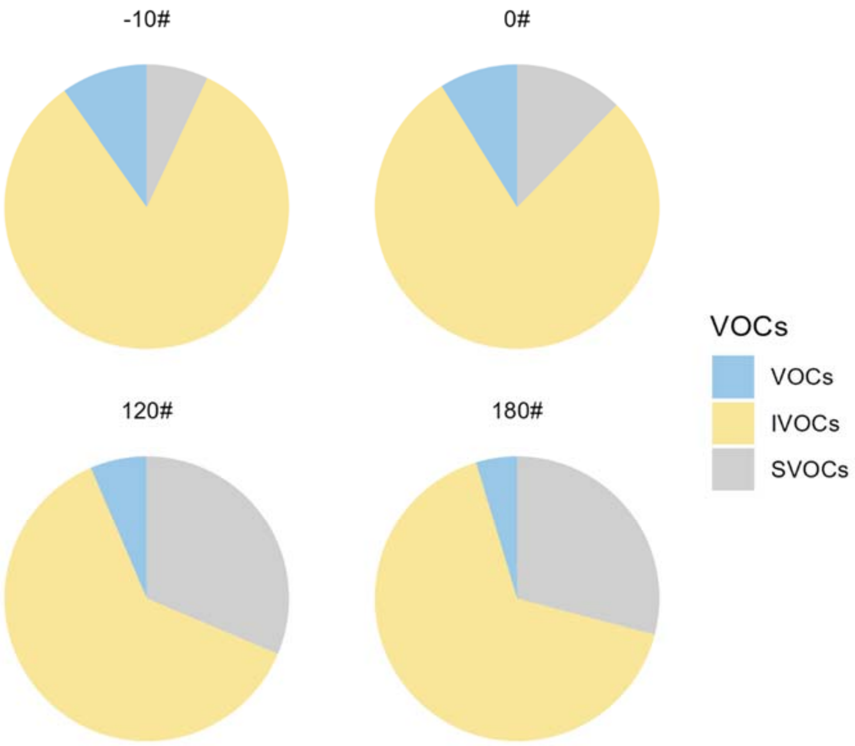

Figure S3. Resolved chemical species in different fuels based on volatilities.
